# Spontaneously Ruptured Hepatocellular Carcinoma: Computed Tomography-Based Assessment

**DOI:** 10.3390/diagnostics13061021

**Published:** 2023-03-07

**Authors:** Fabio Sandomenico, Valerio Arpaia, Ferdinando De Rosa, Orlando Catalano, Roberto Antonino Buonaiuto, Marianna Notarangelo, Maria Iovino, Sabrina Giovine, Arturo Brunetti, Mariano Scaglione

**Affiliations:** 1 Radiology Unit, Buon Consiglio Fatebenefratelli Hospital, 80123 Naples, Italy; 2 Diagnostic Imaging and Radiotherapy Department, Azienda Ospedaliera Universitaria “Federico II”, 80131 Naples, Italy; 3 Department of Radiology, SG Moscati Hospital, ASL Caserta, 81131 Aversa, Italy; 4 Radiology Unit, Istituto Diagnostico Varelli, via Cornelia dei Gracchi 65, 80126 Naples, Italy; 5 Radiology Unit, San Giuliano Hospital, Giugliano in Campania (NA), 28100 Novara, Italy; 6 Department of Surgery, Medicine and Pharmacy, University of Sassari, 07100 Sassari, Italy

**Keywords:** CT, key findings, emergency, hemoperitoneum, hepatocellular carcinoma, HCC, spontaneous rupture, review

## Abstract

Spontaneously ruptured hepatocellular carcinoma (SRHCC) is an uncommon and life-threatening complication in patients with hepatocellular carcinoma (HCC). It is usually associated with chronic liver disease and has a poor prognosis with a high mortality rate during the acute phase. SRHCC can cause a severe and urgent condition of acute abdomen disease and requires a correct diagnosis to achieve adequate treatment. Clinical presentation is related to the presence of hemoperitoneum, and abdominal pain is the most common symptom (66–100% of cases). Although the treatment approach is not unique, trans-arterial (chemo)embolization (TAE/TACE) followed by staged hepatectomy has shown better results in long-term survival. A multi-phase contrast-enhanced CT (CECT) scan is a pivotal technique in the diagnosis of SRHCC due to its diagnostic accuracy and optimal temporal resolution. The correct interpretation of the main CT findings in SRHCC, such as active contrast extravasation and the sentinel clot sign, is fundamental for a prompt and correct diagnosis. Furthermore, CT also plays a role as a post-operative control procedure, especially in patients treated with TAE/TACE. Therefore, a multi-phase CECT scan should be the diagnostic tool of choice in SRHCC since it suggests an immediate need for treatment with a consequent improvement in prognosis.

## 1. Introduction

Hepatocellular carcinoma (HCC) is the most common liver cancer and the sixth most common cancer overall [1]. It is the most frequent cause of death due to cancer worldwide. Spontaneously ruptured hepatocellular carcinoma (SRHCC) is an uncommon and life-threatening complication that usually occurs in tumors at an advanced stage. Its incidence in Asia and Africa is up to 26%, compared with less than 3% in Western countries [2]. This difference is clearly related to the higher prevalence of HCC in developing nations due to higher environmental and infectious exposure and lower healthcare resources [3]. In patients affected by HCC, rupture is the third cause of death after tumor progression and hepatic failure, with a high mortality rate during the acute phase [4,5,6]. According to Moris et al., the mean age for SRHCC is 55.6 years, with a sharp predilection for male patients (77.9%) [2]. Cirrhosis is the most important risk factor for developing HCC since up to 80% of these tumors arise from cirrhotic livers [7,8]. Cirrhosis and other chronic liver diseases can affect the hepatic mechanisms of repair and increase the probability of SRHCC. Other than cirrhosis, the main risk factors for SRHCC are hypertension, extra-hepatic invasion, concomitant ascites, and HCCs with a size >5 cm and/or protruding margins [9]. Even today, the mechanisms of SRHCC are still unclear, and several hypotheses have been made: rapid tumor growth with intra-lesional necrosis, separation between tumor and normal parenchyma, vascular erosion, and increased intra-tumoral pressure due to venous occlusion and coagulopathy [10,11].

Even though correct management of SRHCC is still being discussed, the primary goals of treatment should always be stabilization of the patient and prevention of hypovolemic shock [10]. Analyzing the various findings from past and more recent literature reviews, we emphasized all CT findings indicative of SRHCC by associating them with clinical and pathogenetic data and describing treatment options in order to provide a useful guide to the radiologist in diagnosing this emergency disease.

### 1.1. Risk Factors for SRHCC

SRHCC is a rare complication that occurs mainly in patients with poor liver function, such as in cases of viral hepatitis and cirrhosis. However, other diseases characterized by chronic liver inflammation can cause HCC even in the absence of cirrhosis. Chronic infections from HBV and HCV are the most frequent causes of HCC worldwide and increase the risk of developing tumors even in patients without cirrhosis [3,12,13]. Excessive intake of alcohol increases the risk of HCC since it causes chronic liver disease and cirrhosis, even though tumor incidence is higher in patients with HBV/HCV-related cirrhosis [14,15,16]. HBV causes hepatocellular genomic instability by promoting overexpression of oncogenes and downregulation of tumor suppressor genes. On the other hand, viruses also cause a chronic inflammatory state that promotes liver carcinogenesis [17]. The tumor microenvironment (TME) is generated by uncontrolled interaction between malignant and non-malignant cells: healthy cells stimulate uncontrolled cell proliferation while malignant ones invade normal parenchyma and spread elsewhere via the blood or lymphatic system [18]. Exposure to toxins such as aflatoxin B1 or vinyl chloride causes molecular dysregulation, increasing the incidence of tumors. It has been seen that aflatoxin B1 causes a 4-fold higher risk of HCC than normal, while the risk of HBV infection is 7-fold higher. Nonalcoholic steatohepatitis (NASH) is a more and more emergent cause of chronic liver disease, and several studies demonstrate how NASH-associated HCCs often arise in the absence of cirrhosis [19]. Nonalcoholic fatty liver disease is another condition with a growing impact on the development of HCC in non-cirrhotic patients [20,21,22]. Although the reported association between HCC and obesity is currently quite weak, it is known that obesity is related to a global increase in tumor incidence. Obesity causes significant insulin resistance that contributes to fatty liver disease, liver fibrosis, and subsequent progression to cancer [23]. Several studies have also reported an association between oral contraceptives and the development of HCC. In fact, nuclear overexpression of estrogen receptors has been demonstrated in HCC [24]. Recent studies have stressed how dysbiosis could be involved in liver carcinogenesis by determining an increased transport of toxins to the liver. Dysbiosis stimulates hepatic Kuppfer cells, leading to an increased secretion of pro-inflammatory cytokines (TNF-α, IL-8, and IL-1β). IL-1β increases liver accumulation of lipids by promoting steatosis and cellular programed death. Thus, interleukins promote the progression of NAFLD into NASH and cirrhosis. Increased bacterial translocation could be another risk factor for HCC [25]. Several studies have reported that sterilization of the intestinal microbiota in subjects with advanced HCC resulted in a significant reduction in tumor size, suggesting that the microbiota could be one of the therapeutic targets in the advanced stages of the disease [26,27]. The molecular mechanisms of HCC are driven by reactivation of the Wnt/β-catenin pathway, which regulates hepatobiliary differentiation during embryogenesis and hepatic homeostasis in adulthood. In about 25–30% of patients, this pathway is hyperactivated, probably due to epigenetic modifications [28]. Long non-coding RNAs (lncRNAs) from ultra-conserved regions seem to be involved in the reactivation of the Wnt/β-catenin pathway and thus in genesis of HCC [29]. The Wnt/β-catenin pathway promotes overexpression of genes involved in cellular proliferation. Abnormalities in this pathway determine the development and progression of several liver diseases, such as hepatic fibrosis, steatosis, cholestasis, HCC, cholangiocarcinoma, and hepatoblastoma [30,31].

Although many hypotheses have been proposed through the years, the mechanisms of SRHCC are still debated. Cirrhosis, hypertension, ascites, a high Child-Pugh score, an advanced BCLC stage, and a large tumor size are considered the main predictors for SRHCC [32,33,34]. Cirrhosis (and other chronic liver diseases) is one of the main predisposing conditions since it makes the parenchyma more friable and affects the production of hepatic coagulation factors [6]. It seems acquired that rupture occurs more easily in tumors with large dimensions (>5–7 cm) and/or with margins protruding from the hepatic surface [9,10]. However, spontaneous rupture of smaller HCCs (about 2 cm) is not so rare [35], suggesting that other underlying conditions could be involved. Mechanical factors associated with increased intra-tumoral pressure could play a crucial role. Therefore, HCCs rich in necrosis, in rapid growth, or accompanied by vascular congestion could result in more spontaneous rupture [5]. The presence of peri-hepatic ascitic fluid can sometimes cause increased pressure in the hepato-diaphragmatic space and laceration of the tumoral surface and adjacent arteries. According to the “small room hypothesis”, HCCs arising from smaller segments (I, II, III, or VI) could rupture more frequently since these segments undergo a higher pressure from the liver capsule [10,36]. Instead, tumors arising from larger segments could be more protected from rupture due to a greater quantity of normal surrounding parenchyma. Vascular dysfunction is another factor associated with SRHCC. In patients affected by chronic liver disease, inflammation causes vascular damage due to increased production of collagenase [6,10]. In some cases, chronic vasculopathy could make vessels stiffer with a loss of elasticity and increase the risk of rupture and bleeding. In patients with HBV, vascular injury is caused by the deposition of antigen-antibody complexes that are not effectively removed by liver macrophages [6].

### 1.2. Clinical Presentation of SRHCC

SRHCC is usually accompanied by the following manifestations:

(a) sudden-onset epigastric pain;

(b) severe hypotension; 

(c) drop in hemoglobin concentration.

Clinical manifestations of SRHCC are related to anemia and peritoneal reactions due to the presence of hemoperitoneum. Sometimes it has a subclinical development with slow hemorrhage and progressive anemization. Instead, in cases of massive hemoperitoneum, it usually has a rapid onset with an elevated risk of mortality. Abdominal pain is the most common symptom (66–100% of the cases) and is often accompanied by abdominal distension (33% of the cases) [10]. In some cases, peritoneal reactions could not be present, especially in HCC with central localization [37]. Progressive anemia is typically associated with paleness, dizziness, fatigue, palpitations, and shortness of breath, and it can worsen until a critical condition of hypovolemic shock [6]. Concomitant jaundice and ascites are also frequent due to the strong association between HCC and cirrhosis. Several studies have reported liver failure during the acute phase in about 12–42% of patients [4,10].

### 1.3. Diagnosis of SRHCC

Imaging techniques are essential for the management of HCC, and a conclusive diagnosis is often made by imaging alone without the need for a histological examination [38]. Ultrasonography (US) is widely used for screening and plays a role in emergency situations, even in traumatic injuries [39,40]. More invasive procedures such as contrast-enhanced ultrasonography (CEUS), multi-phase contrast enhanced computed tomography (CECT), and magnetic resonance imaging (MRI) are recommended for a conclusive diagnosis of HCC [39]. Among systems for the correct management of HCC, the *Liver Imaging Reporting and Data System (LI-RADS**^®^***) is the most complete guide for a proper diagnosis [41]. The LI-RADS**^®^** system is mainly composed of 5 categories (from LR-1 to LR-5). LR-1 indicates absolute benignity, while LR-5 indicates the presence of HCC with a diagnostic specificity of 95%. In addition, there are also three additional categories: LR-NC (uncategorizable), LR-TIV (tumor in vein), and LR-M (malignant or probably malignant tumor) [42,43]. LI-RADS**^®^** categories are assigned based on the observation of five major imaging features and other secondary features. The main ones include non-rim arterial hyperenanchment (APHE), non-peripheral washout, capsule enhancement, tumor size, and margin growth. Ancillary features (FAs) are divided into three groups: FAs suggestive of malignancy in general, FAs suggesting HCC, and FAs in favor of benignity [44]. LI-RADS**^®^** provides a standardized modality of imaging management with the application of this flowchart: (a) ultrasound for screening and surveillance; (b) CEUS, CECT, or MRI for diagnosis, staging, and evaluation of treatment and possible complications [45,46].

SRHCC is a rare condition, and a traumatic cause, even from a slight trauma, should always be suspected [47]. However, correct management of patients with HCC should always consider the risk of SRHCC. A prompt diagnosis is required to achieve adequate treatment.

#### 1.3.1. Ultrasonography (US)

The US diagnosis of a HCC is based on the shape, size, margins, or inhomogeneity of the echogenicity of suspect lesions. HCCs present themselves as hypoechoic (23–54%), hyperechoic (12–38%), or mosaic-patterned (17–38%). Nodules smaller than 10 mm are usually hypoechoic, while larger lesions are more frequently hyperechoic or mosaic-patterned. The most frequent US findings of HCC are: (a) posterior hyperechogenicity; (b) lateral shadow sign; and (c) halo sign (when a fibrous capsule is present) [48].

US is a first-level procedure in emergencies and depicts the site of rupture as a bright area nearby a tumor in 66% of cases [49]. However, US has shown some limitations in patients with small-sized tumors [50,51]. CEUS is another useful test since it has an elevated resolution and shows in real time hemorrhage from spontaneous or non-spontaneous rupture [52]. It usually displays HCC as contrast-enhancing lesions with quick, subsequent washout [53]. CEUS washout is defined as a partial or total reduction of echogenicity compared to normal liver parenchyma [54]. In some cases, HCCs can show inhomogeneous contrast enhancement due to intra-lesional necrosis or peripheral hypervascularity in the presence of the tumor capsule. Several studies have demonstrated that active extravasation of blood can also be seen with CEUS and commonly presents itself either as a “puddle” or “jet” leak [55]. In general, CEUS depicts bleeding with two different patterns: jet-like extravasation or blister leakage. Traumatic tumoral ruptures could show a different US pattern from SRHCC [56].

#### 1.3.2. Computed Tomography (CT)

The helical multi-phase CECT scan, for its elevated spatial resolution and speed of execution, is a prompt procedure and has a pivotal role in the diagnosis of SRHCC. CT has shown a diagnostic accuracy of 75–100%, avoiding the necessity of more invasive procedures such as angiography or paracentesis [6]. CT also plays a role in the control of ruptured HCCs treated with chemoembolization. In fact, it demonstrates the success of this procedure by showing the presence of a hyperdense drug (Lipiodol) near the tumor and the consequent interruption of intra-tumoral blood flow. The CT diagnosis of SRHCC is treated accurately in the next main paragraph below (see Section 2).

#### 1.3.3. Magnetic Resonance Imaging (MRI)

Although MRI is a very useful technique in the diagnosis of HCC, it plays a limited role in the case of SRHCC, mainly due to its elevated temporal resolution.

### 1.4. Treatment of SRHCC

The treatment approach for SRHCC is not unique and must be evaluated case by case. The main current strategies are conservative treatment, hepatic resection, trans-arterial (chemo)embolization (TAE/TACE), or a combination of TAE/TACE + hepatic resection. SRHCC usually occurs in hemodynamically unstable patients due to their poor liver function. Therefore, hemostasis should always be the first goal to be pursued. Conservative treatment consists of continuous monitoring, control of hemostasis, fluid infusion, and nutritional support [6]. However, it has shown poor results and should be considered only in terminal patients or in stable subjects with minor ruptures. Emergency hepatic resection has been the main approach for years, with good results in the short and medium terms. It has the advantage of achieving both definitive treatment and efficient hemostasis [57]. However, this treatment is not recommended in unstable patients for its high risk of peri-procedural mortality [57]. Elective hepatectomy has shown more favorable results than emergency hepatectomy, although it has an increased risk of intra-peritoneal tumor dissemination. Therefore, early partial hepatectomy seems to be the best approach since it has a lower mortality rate, allows a correct classification of pre-operative patients, and has a lower or equal dissemination rate than emergency procedures [58]. Interventional treatment is essential to achieve hemostasis rapidly and provides a good substrate for subsequent surgery. Different types of embolizing agents can be chosen depending on tumor location, degree of bleeding, and experience of the operator. Iodized oil (LipiodolVR; Guerbet, Villepinte, France) mixed with an anti-cancer substance and gelatin sponge (GS) (Gelfoam; Upjohn, Kalamazoo, MI) is the most widely used agent [59]. However, efficacy and safety of embolizing agents are still seldom discussed in the literature [60]. TACE is a minimally invasive technique that has shown many advantages compared to a classic hepatectomy. It is performed under local anesthesia and allows excellent visualization of the arteries supplying the tumor that must be embolized. In some cases, HCC can recruit extra-hepatic collateral vessels such as the gastroduodenal and superior mesenteric adrenal arteries, resulting in incomplete embolization and bleeding recurrence [61]. Furthermore, patients treated with TACE could undergo post-chemoembolization syndrome with widespread pain, myalgias, and fever. Cases of post-procedural acute kidney injury have also been described [62,63]. Recent studies have demonstrated how combined treatment achieves both effective hemostasis and a lower risk of tumoral recurrence [64].

Palliative treatments with the sole purpose of hemostasis have also been proposed: (a) ligation of the hepatic artery; (b) suture of the bleeding tumor; (c) microwave or radiofrequency ablation [5].

### 1.5. Prognosis of SRHCC

As stated before, tumoral rupture is the third cause of death related to complications of HCC [4]. SRHCCs carry a worse prognosis than non-ruptured lesions [65]. Patient survival is closely related to the type of therapy performed. Previous studies have reported a mortality rate in the acute phase of 25–75% [4,5,6]. Although survival has shown an increase over the past few years, there are still many deaths among patients with advanced tumors. One reason is that these patients are not always evaluated and treated in specialized healthcare structures, especially in developing countries. Prognosis is extremely poor in untreated patients, with a reported median survival of less than 4 months [66]. In addition, the survival rate for patients undergoing conservative therapy is extremely low. According to a study by Zhong et al., including 162 patients treated conservatively, the survival rate for the 30-day period has been 8.6%, and there have been no survivors at the 1-year mark [67]. Hsueh et al. stressed how conservative therapy results as an independent predictor of poor long-term survival [68]. Several studies have demonstrated a significant decrease in mortality overall (23.5%) owing to the excellent outcome of patients treated with hepatectomy (<1%) [32]. Lai et al. have reported survival rates using hepatic resection at the 1- and 5-year endpoints of 76.0% and 33.9%, respectively. On the other hand, the reported survival rates with staged hepatectomy have been 90% and 67.5% at 1 and 5 years, respectively [5]. Anyway, Xu et al. recently stressed that SRHCC is a risk factor for long-term prognosis even in cases of successful hepatic resection [69]. Recent studies have reported a similar outcome for TAE/TACE compared to emergency resection at the 1-year mark. However, TAE/TACE has shown itself to have superior efficacy in terms of in-hospital survival and therefore should be preferred as an emergency treatment for SRHCC [70,71]. Furthermore, staged hepatectomy has shown better results in long-term prognosis compared to emergency resection [72]. Currently, TACE followed by staged hepatectomy has shown better results in long-term survival than hepatectomy or TACE alone and is now considered the most effective treatment [6,73,74,75,76,77].

## 2. CT Findings

### 2.1. Differential Diagnosis of HCC

HCC is a relatively common tumor in cirrhotic subjects, and about 1–8% of these patients receive a new diagnosis of HCC every year. The CECT scan shows a high sensitivity for the diagnosis of HCC, even in cases of small tumors [78]. Radiologists should always achieve an early diagnosis and differentiate tumors from other common liver findings related to cirrhosis [79]. HCC has a typical enhancement pattern that allows differentiating malignant tissue from normal liver parenchyma. It is a hypervascular tumor, and therefore it shows an intense contrast enhancement during the arterial phase and subsequent wash-out in the portal and delayed phases, in which it appears hypodense [7,80,81]. HCC must be differentiated from other lesions such as regenerative cirrhotic nodules, arterioportal shunts (APSs), hepatic hemangiomas, pseudomasses, focal fatty changes, and focal nodular hyperplasia-like lesions [82]. Main CT findings of these lesions allow making a differential diagnosis with HCC:

- Regenerative nodules are isodense to liver parenchyma with absent wash-in during the arterial phase. The presence of surrounding fibrosis can be evaluated during the portal phase [83].
- Arterioportal shunts (APSs) are abnormal communications between a hepatic arterial branch and the corresponding portal vein, sinusoid, or peri-biliary venule, causing focal arterialization of liver parenchyma. APSs are observed in the arterial phase as peripheral wedge lesions that become isodense to liver parenchyma during the portal and late phases [84].
- Hepatic hemangiomas show peripheral, globular, and centripetal enhancement during the dynamic phases of contrast-enhanced CT [85].
- Pseudomasses are commonly observed in chronic portal vein thrombosis due to changes in the liver parenchyma from abnormal blood flow. Pseudomasses have a hypertrophic central area that shows hyperdensity in the arterial phase and appears isodense to liver parenchyma during the delayed phase. Instead, the peripheral region is usually atrophic and shows itself as constantly hypodense [86].
- Large regenerative nodules (LRNs), also known as focal nodular hyperplasia-like lesions, are clustered regenerative nodules showing rapid, intense, and persistent wash-in during the arterial phase with absent wash-out in the portal and delayed phases [87].

### 2.2. Spontaneously Ruptured HCC (SRHCC)

A multi-phase contrast-enhanced CT (CECT) scan is the diagnostic procedure of choice in SRHCC due to its optimal spatial and temporal resolution. The study of the liver with its focal lesions and the presence of hemorrhage should be key points to consider for a correct CT diagnosis. CT scanning identifies tumoral position and allows for the prediction of rupture risk in lesions with larger dimensions and/or protruding margins. Hemoperitoneum could be visible with an unenhanced CT scan [88]. However, recent studies have demonstrated how unenhanced CT does not improve the diagnosis of hemoperitoneum and could expose patients to an unnecessary radiation dose [89]. The multi-phase protocol gives more information about tumoral vascularization and has a key role in the identification of active hemorrhage [90,91]. Therefore, a CECT scan is the best procedure to identify intra-abdominal hemorrhage, especially when the bleeding rate is low. Intra-peritoneal blood reaches the declivous spaces, and the supine decubitus assumed for CT examination influences its disposition. Pouches of Morrison (hepato-renal space) and Douglas (recto-uterine/recto-vesical space) are the most declivous areas in supine decubitus. In the presence of bleeding SRHCC, hemorrhage first involves the hepato-renal space and then the pelvic cul-de-sac by crossing the para-colic spaces [47]. In patients with cirrhosis and ascites, CT displays hemorrhagic contamination of ascitic effusion since blood has a higher density than other fluids. Accumulation and subsequent degradation of hemoglobin can cause a time-dependent variation in density values. At an unenhanced CT scan, hemorrhage has values of 30–45 HU during the hyperacute phase. After a few hours from rupture (24–72 h), blood shows increased values (>60 HU) due to coagulation and accumulation of hemoglobin [92]. After 10–30 days, hematomas show a progressive decrease in density with the development of an external pseudocapsule [93].

The main CT signs of SRHCC are treated separately below.

**Figure 1:**
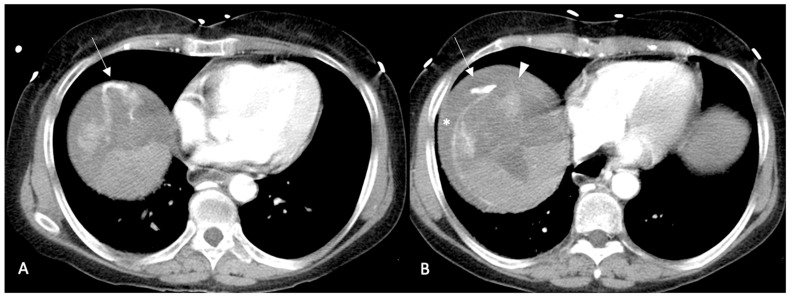
Arterial phase enhanced CT scan. HCC of the VII–VIII segment with intra- and extracapsular hemorrhage and active contrast extravasation in the context of peritoneal effusion ((**A**,**B**) arrows). (**B**) Interruption of the Glissonian capsule (arrowhead) with concomitant hemoperitoneum (asterisk) is clearly visible.

**Figure 2:**
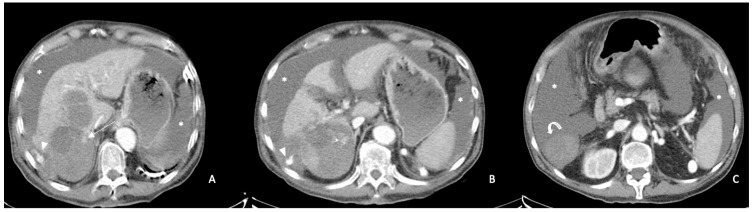
Arterial phase enhanced CT scan. HCC of the VII segment with necrotic intra-tumoral areas (arrows) and concomitant peri-hepatic and peri-splenic effusions (asterisks). Active contrast extravasation is visible near the tumor surface inside the hematoma (arrowheads (**A**,**B**)). The sentinel clot sign is also visible ((**C**), curved arrow).

***Active contrast extravasation (*Figure 1 and Figure 2*).*** Multi-phase CECT can show active extravasation of contrast medium that is highly suggestive of acute hemoperitoneum and stresses the necessity for an immediate treatment (TAE/TACE or surgical resection) [88]. In patients with suspected hemorrhage, a hyperdense focus extending into a hyperdense collection is highly suggestive of contrast extravasation. In patients with large hemorrhages or low cardiac output, arterial phase sequences may not display active extravasation, and active leaks could be demonstrated only in portal and/or delayed phase sequences [94,95].

**Figure 3:**
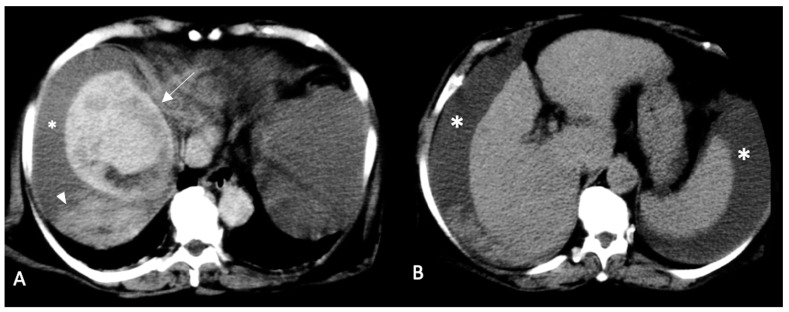
Multi-phase enhanced CT scan (portal phase (**A**) and late phase (**B**)). Large HCC of VII-VIII segment with intracapsular hemorrhage and sentinel clot (arrow). Peri-hepatic and peri-splenic ascites effusions are also visible (asterisks). Perihepatic fluid appears to be contaminated by blood in the dependent site (hematocrit sign, arrowhead (**A**)).

**Figure 4:**
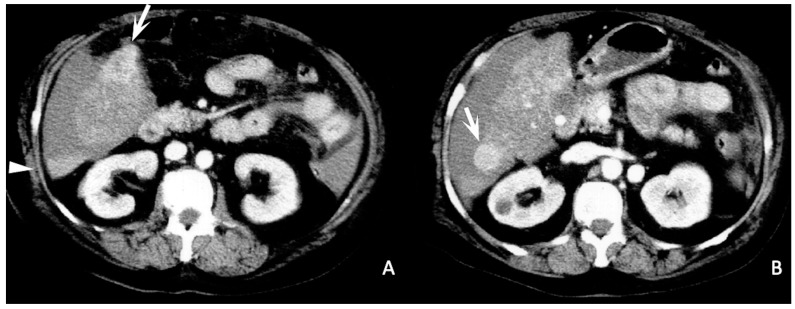
Arterial phase enhanced CT scan. Subcapsular HCCs (arrows (**A**,**B**)) surrounded by peri-hepatic hematoma. A double fluid-fluid level with a hyperdense clot in dependent position is clearly visible (the hematocrit sign and the sentinel clot sign) (arrowheads (**A**)).

***Sentinel clot sign (***Figure 2, Figure 3 and Figure 4***).*** This sign is one of the most suggestive findings of organ injury and has a role in the diagnosis of occult hemorrhages [96]. Blood with the highest density is commonly seen nearby the hemorrhage site, while less attenuating portions of hematoma are observed more distally. Recent clots have a higher attenuation value than non-coagulated blood or chronic hemorrhage. The presence of a clot with a higher density near the tumoral site identifies the initial hemorrhagic focus [96]. This sign is also useful for undiagnosed small tumors and is similar to findings described in visceral traumas [97].

**Figure 5:**
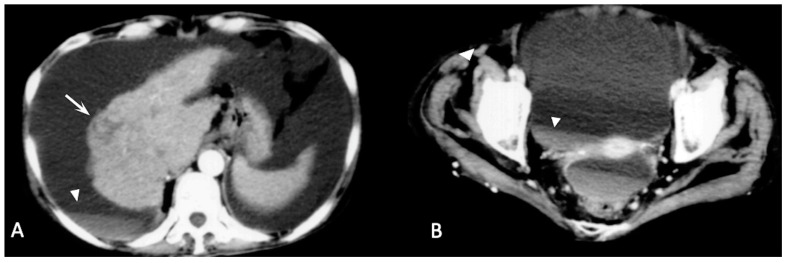
Arterial-phase enhanced CT scan. Protruding subcapsular HCC of the VIII segment with intra-tumoral necrosis and a slight enucleation sign (arrow). Peri-hepatic and peri-splenic effusions are also visible. Peri-hepatic fluid is contaminated by blood in the dependent site (hematocrit sign, arrowheads (**A**,**B**)).

**Figure 6:**
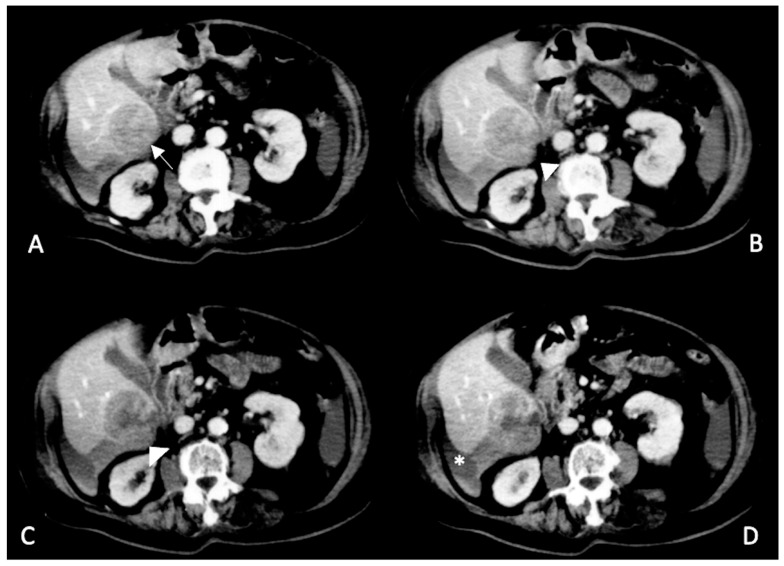
Arterial-phase enhanced CT scan. (**A**) Capsulated HCC of the VIII–V segment with inhomogeneous density (arrow). Note the disruption of the capsule with the consequent emptying of tumoral content (enucleation sign, arrowheads (**B**,**C**)). The hematocrit sign is also present ((**D**), asterisk).

***Enucleation sign (*Figure 5 and Figure 6*).*** This sign has been called this owing to its similarity to an enucleated orbital globe with residual peripheral sclera. It is visible in contrast-enhanced sequences and depicts the tumor as an unenhancing hypodense focus surrounded by a hyperdensely enhanced rim due to the emptying of its content into the peri-hepatic space [10,98]. The peripheral enhancing border is not part of the tumor and consists of normal compressed parenchyma [10]. When accompanied by the presence of surrounding hematoma and/or active contrast extravasation, this finding is highly specific for SRHCC [10].

***Hematocrit sign (*Figure 3, Figure 4, Figure 5 and Figure 6*).*** This sign is visible with unenhanced CT and is due to the process of separation of various blood components. Blood is rich in proteins, and therefore its density is usually around 35–40 HU. Coagulated blood shows even higher density (>60 HU). In some cases, separation between various components of the hematoma determines the presence of a double fluid-fluid level with different attenuation values, of which the more dependent one is spontaneously hyperdense (hematocrit effect) [92,99]. In patients with advanced liver disease and subcapsular HCC, this finding could lead to the diagnosis of hemoperitoneum even with minimal extravasation.

***Pseudo-retraction sign***. In some cases, the presence of hematomas causes deformation of the tumoral profile and simulates a false capsular retraction [100]. In the presence of large peripheral HCCs accompanied by a small surrounding collection, this finding shows a high sensitivity for the diagnosis of confined ruptures [37].

## 3. Conclusions

SRHCC can cause a severe and urgent condition of acute abdominal disease that requires a prompt and correct diagnosis. As an initial step, the main goals of treatment must be effective hemostasis and stabilization of all parameters with infusive support and/or emergency procedures such as TAE/ TACE, or liver resection. Afterwards, in stable subjects, a staged hepatectomy can be performed to achieve conclusive therapy. Although several studies have focused on the application of CEUS for the identification of this rare complication, it has shown some limitations in the emergency setting. Multi-phase CECT, due to its optimal spatial and temporal resolution, plays a crucial role in the management of this complication. The CECT predicts tumor rupture by identifying the location and size of the culprit lesions. Active extravasation of contrast medium, the sentinel clot sign, the enucleation sign, the hematocrit sign, and capsular pseudo-retraction are the key points to bear in mind for an imaging diagnosis. Furthermore, CT readily identifies acute hemoperitoneum, suggesting an immediate need for treatment with a consequent improvement in prognosis.

## Data Availability

Not applicable.
